# SHORT-TERM EFFECT OF WHEY PROTEIN SUPPLEMENTATION ON THE QUALITY OF LIFE OF PATIENTS WAITING FOR LIVER TRANSPLANTATION: A DOUBLE BLINDED RANDOMIZED CLINICAL TRIAL

**DOI:** 10.1590/0102-672020210002e1596

**Published:** 2021-10-18

**Authors:** Michelle Carvalho de Oliveira D’ALESSANDRO, Amanda Dias GOMES, Jéssica França MORAIS, Yani Glaúcia Gomide MIZUBUTI, Thales Antônio da SILVA, Silvia Mauricio FERNANDES, Larissa Loures MENDES, Maria Isabel Toulson Davisson CORREIA, Simone de Vasconcelos GENEROSO

**Affiliations:** 1 Nursing School, Department of Nutrition/Nutrition and Health Program, Federal University of Minas Gerais, Belo Horizonte, MG, Brazil; 2 Pharmacy School/Food of Science Program, Federal University of Minas Gerais, Belo Horizonte, MG, Brazil; 3 Faculty of Medicine, Department of Surgery/Surgery and Ophthalmology Program, Federal University of Minas Gerais, Belo Horizonte, MG, Brazil

**Keywords:** Quality of life, Whey protein, Nutritional status, Liver transplantation, Qualidade de vida, Proteína do soro do leite, Estado nutricional, Transplante hepático

## Abstract

**Background:**

Chronic liver disease is associated with malnutrition that negatively impacts a patient’s health-related quality of life (HRQoL).

**Aim:**

To evaluate the short-term effect of whey protein supplementation on the HRQoL and nutritional and functional status of patients waiting for liver transplantation.

**Methods:**

This was a double-blind randomized clinical trial with patients waiting for liver transplantation who were randomized into two groups: WP (whey protein supplementation) and the control (casein supplementation). Both groups received 40 g (20 g in the morning and 20 g in the evening) for 15 days. Nutritional and functional status were evaluated. Energy balance was calculated as the difference between energy intake (24-hour recall) and total energy expenditure (assessed by indirect calorimetry). The chronic liver disease questionnaire was used to assess HRQoL. All measurements were performed before and after the intervention.

**Results:**

Fifty-six patients were evaluated. Malnutrition was present in 56.9%, and it was directly associated with a poor HRQoL (p<0.05). No improvement on the nutritional and functional status was observed, in either group after protein supplementation. HRQoL improved after WP and casein supplementation, with no differences between groups (p>0.05). Patients who met protein requirements and had a positive energy balance demonstrated a higher HRQoL score (4.9, p<0.05), without between-group differences.

**Conclusion:**

Malnutrition substantially reduces HRQoL. Short-term WP or casein supplementation improved similarly the HRQoL.

## RESUMO

**Racional:**

A doença hepática crônica está associada à desnutrição que afeta negativamente a qualidade de vida relacionada à saúde (QVRS).

**Objetivo:**

Avaliar o efeito da suplementação aguda de proteína do soro de leite na QVRS e no estado nutricional e funcional dos pacientes em lista de espera para o transplante hepático.

**Métodos:**

Ensaio clínico randomizado com pacientes à espera do transplante hepático que foram randomizados em dois grupos: PS (suplementação com proteína do soro de leite) e Controle (suplementação com caseína). Ambos os grupos receberam 40 g das proteínas (20 g pela manhã e 20 g à noite) por 15 dias. O estado nutricional e funcional foi avaliado. O balanço energético foi calculado como a diferença entre a ingestão energética (recordatório de 24 horas) e o gasto energético total (avaliado por calorimetria indireta). O questionário de doença hepática crônica (CDLQ) foi utilizado para avaliar a QVRS. Todas as medidas foram realizadas antes e após a intervenção.

**Resultados:**

Cinquenta e seis pacientes foram avaliados. A desnutrição esteve presente em 56,9% e, diretamente associada à baixa QVRS (p<0,05). A suplementação, com ambas as proteínas, não alterou o estado nutricional e funcional dos pacientes (p>0,05). Entretanto, a QVRS melhorou após a suplementação com PS e caseína, sem diferenças entre os grupos (p>0,05). Pacientes que alcançaram as necessidades proteicas e com balanço energético positivo tiveram maior escore de QVRS (4,9; p<0,05), sem diferenças entre os grupos (p>0,05).

**Conclusão:**

A desnutrição reduz substancialmente a QVRS. A suplementação aguda com PS ou caseína melhorou de forma similar a QVRS.

## INTRODUCTION

Liver transplantation (LT) is the only treatment for patients with end-stage liver disease and acute liver failure ^[^ ^1^ ^]^, offering them the chance to increase their life expectancy, and often leading to a better quality of life ^[^ ^2^ ^]^. However, long waiting list time until the operation, particularly in developing countries, negatively impacts patients’ quality of life^[^ ^3^ ^]^ and their overall clinical status. These patients commonly present several complications associated with the evolution of liver disease, such as anorexia, early satiety due ascites and portal hypertension, asthenia, esophageal varices, and hepatic encephalopathy^[^ ^4^ ^]^, which interfere with their nutritional and functional status, and thus with their health-related quality of life (HRQoL)^[^ ^5^ ^]^. 

HRQoL refers to the results of subjective and dynamic assessments of a patient’s self-perception of state of health. It integrates physical, mental, and social contexts related to health^[^ ^6^ ^]^, in addition to changes in functional capacity, daily activities, and emotional relationships in the patient’s life that can cause a decrease in HRQoL^[^ ^7^ ^]^.

Previous studies have shown that the quality of life of patients with cirrhosis is significantly lower than that of healthy individuals^[^ ^8^ ^-^ ^11^ ^]^. These findings have been associated with the severity of liver disease and vary according to changes in the patients’ clinical and nutritional status. They also worsen the functional capacity, leading to impairment of daily living activities and HRQoL. 

Considering the relationship between malnutrition and HRQoL, nutritional interventions should be mandatory as an integral therapeutic approach for liver transplant patients^[^ ^8^ ^,^ ^9^ ^]^. Nutritional therapy, focused on the ingestion of an adequate amount of protein, has been associated with improved liver function and increased nutritional and functional status, and thus a consequent improvement in the quality of life of chronic liver disease and cirrhosis patients^[12, 13]^. Besides increasing the amount of protein, it is essential to consider the quality of protein to promote adequate muscle synthesis^[^ ^10^ ^]^. In this regard, supplementation of high-quality protein such as milk proteins (casein or whey protein), might be an interesting strategy, as they provide essential amino acids directly involved with muscle protein synthesis, consequently improving the overall nutritional status ^[^ ^14^ ^]^. 

This study aimed to evaluate the short-term effect of whey protein supplementation on the HRQoL and nutritional and functional status of patients awaiting liver transplantation.

## METHODS

This was a double-blind randomized clinical trial, carried out with patients awaiting liver transplantation at a public hospital. Patients of both genders, aged ≥18 years, and who were active on the waiting list for LT were invited to participate. Pregnant or breastfeeding women, as well as individuals with advanced kidney disease and those awaiting retransplantation were excluded. The study was conducted according to the Declaration of Helsinki and was approved by the local University Ethics committee (CAAE-27430714.8.0000.5149) and registered at Clinical Trials (NCT02901119). All patients gave written informed consent. 

They were randomly assigned in a 1:1 ratio by computer-generated random numbers into two groups: intervention (WP) and control (C) based on the source of protein supplementation, that is, whey protein or casein, respectively. Participants were assessed by the researchers at two different times: a) first evaluation - full clinical history, nutritional and functional evaluations, 24-hour recall, resting energy expenditure assessment, and quality of life questionnaire. Further, in this first assessment, patients received the supplements and orientation on how to prepare and consume them; b) second evaluation - 15 days after the first one - the patients underwent the same assessments as in the initial moment, and 24-hour recall was collected. Their supplement intake was monitored through weekly telephone calls, and they were instructed to bring the empty packages of supplements on the return visit. Each patient received 30 sachets containing 20 g of whey protein or casein, in each unit, to be taken twice a day, one in the morning and one in the evening, diluted in 150 ml of water or juice. The patients were instructed to maintain consistent eating and living routines during the study. Whey protein and casein sachets appeared similar. The patients and researchers were blinded to the intervention.

The nutritional status was assessed by Subjective Global Assessment (SGA). Patients were classified as nourished, suspected/moderately malnourished, or severely malnourished. For statistical purposes, nutritional status was categorized into two groups: nourished and malnourished (suspected/moderate and severe). Anthropometric evaluation included measurement of the triceps skinfold thickness with a Lange Skinfold Caliper (Cambridge Scientific Industries Inc., Cambridge, MD, USA), the arm circumference, with an inextensible tape, and both were used to calculate the arm muscle area^[^ ^15^ ^]^. The anthropometric measurements were classified according to Frisancho^[^ ^16^ ^]^. The measured values of them below the 5^th^ percentile were considered malnutrition ^[^ ^16^ ^]^. Only one investigator performed the measurements to minimize practical variability, and the average of three consecutive measurements was recorded. 

Muscle functional status was evaluated by handgrip dynamometry (Jamar Plus+^®^) according to the established protocol by Budziareck et al.(2008) ^[^ ^17^ ^]^. Assessments were performed in triplicate, and the average value was used (^[^ ^17^ ^]^, with values below the 5^th^ percentile considered to indicate malnutrition. The 6-minute walking test was performed according to the American Thoracic Society guidelines ^[^ ^18^ ^]^ . 

Resting energy expenditure was measured by indirect calorimetry using the Quark RMR device (Cosmed, Rome, Italy). The test was performed in a silent, temperature-controlled room (22-24° C) in the morning. The patients fasted for 12 h and remained recumbent for approximately 20 min before beginning the test. The total energy expenditure was calculated by adding 20% to the value to it (^[19, 20]^.

Quantitative food intake data were obtained using the 24-hour recall. Diet Pro4R^®^ software (Agromidia Software, Viçosa, Brazil) was used to calculate daily energy intake, proteins, carbohydrates, and lipids. Protein intake was considered adequate for the minimal amounts of 1.2 g/kg of dry body weight ^[^ ^20^ ^]^. Energy balance was calculated as the difference between energy intake obtained in the 24-hour recall and total energy expenditure (EB=EI-TEE); an energy balance below zero was considered negative. 

The HRQoL was evaluated by the Chronic Liver Disease Questionnaire (CLDQ). This is a short instrument consisting of 29 questions distributed in six domains: abdominal symptoms, fatigue, systemic symptoms, activity, emotional function and worry, and each domain has seven levels of responses: from 0 (all time) to 6 (never). The score in each domain was obtained by the sum of the answers and divided by the number of questions answered. These data were classified according to Souza et al. 2015^[^ ^21^ ^]^. Low HRQoL was designated when the total CLDQ score was <5, and high HRQoL when ≥5. 

### Statistical analysis

The study sample size was based on the study by Ong., et al., 2011^[^ ^22^ ^]^ who showed patients with chronic liver disease had an improved of 1.51 points in the CLQD score after the intervention with branched-chain amino acids. Thus, our estimated sample size was 42 patients (21 patients for each group), considering a power of 80%, an alpha of 0.05 and 30.0% of loss of follow up. Frequency distributions, measures of central tendency and dispersion were calculated, and the Shapiro-Wilk test was used to verify the normality of the quantitative variables. The t Student and Wilcoxon tests were used for intra-group comparisons of means and medians, respectively, to evaluate the effectiveness of the intervention. Categorical data were compared using the Chi-square test or Fisher’s test when appropriate. A significance level of 5% was adopted for all analyses. The Stata Statistical Software, version 12.0 was used.

## RESULTS

**FIGURE 1:**
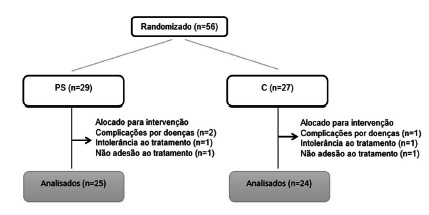
Flowchart of the participants during the phases of recruitment, randomization and intervention

Fifty-six patients were recruited and randomized into the WP and C groups. Seven patients dropped out during treatment (Figure 1). 

WP=whey protein group; C=casein group

There was no difference between the groups in terms of the general characteristics (Table1) and nutritional status at baseline (p>0.05). According to Subjective Global Assessment (AGS), 48.0% and 54.0% of patients were malnourished in the WP and C groups, respectively. There were no significant differences considering dynamometry (WP group: 29.53±1.69 Kg; C group: 31.06±1.64 Kg; p>0.05) and the 6MWT (WP group: 446.0±20.7 meters; C group: 454.6±19.6 meters; p>0.05).

**TABLE 1:** Characterization of the patients at baseline

Characteristics	WP (n=25)	C (n=24)	p
Age (years)	53.3±10.2	50. 4±12.4	>0.05 γ
CLD etiology (n, %) Ethanolic	8.0 (32.0%)	5.0 (20.8%)	> 0.05 ^ɫ^
Virus B and C	10.0 (40.0%)	12.0 (50.0%)	>0.05 ^ɫ^
Other	7.0 (28.0%)	7.0 (29.2%)	>0.05 ^ɫ^
MELD	15.3±0.7	15.6±0.8	>0.05*
Edema (n, %)	16.0 (64.0%)	16.0 (66.6%)	>0.05 ^ɫ^
Ascites (n, %)	12.0 (54.5%)	18.0 (75.0%)	>0.05 ^ɫ^
Medications ≥3 (n, %)	21.0 (84.0%)	21.0 (87.5%)	>0.05 ^ɫ^

* t test; γ Wilcoxon; ^ɫ^ chi-square WP=whey protein group; C=casein group**;** CLD=Chronic Liver Disease; MELD=Model for End-Stage Liver Disease

Energy (20.2 vs. 23.0 kcal/kg) and protein (0.8 vs. 0.9 g/kg) intake were similar between the groups before the protein supplementation (p>0.05), with 78.6% and 73.1% of the patients in the WP and C group, respectively, eating less than the recommended protein allowances. The mean energy balance was -558.7±639.7 kcal in the WP group and -413.2±504.4 kcal in the C group (p>0.05). 

At the beginning of the study, the patients HRQoL was similar between the groups. The total CLDQ score was 4.0 for the WP group and 3.8 for the C group (p>0.05), with only 10.3% of the patients presenting a total score over 5.0, prior to intervention. Only nutrition status was associated with poor HRQoL (p<0.05). Among those individuals who had HRQoL<5, 64.7% were classified as moderately or severely malnourished. Neither the severity of the disease (indicated by the MELD score) nor the functional status influenced the HRQoL of patients (p>0.05).

Patients in both groups presented an increased quality of life after the intervention. The CLDQ score increased by 0.97 points (3.73±1.03 before the intervention and 4.70±0.68 after the intervention, p<0.05). After the intervention, 47.8% presented a total score over 5.0. However, there were no significant differences between the groups (p>0.05). Considering each CLDQ domain, most scores increased after the protein supplementation compared to the baseline (p<0.05), except for the domains of abdominal symptoms (p>0.05, Table 2). The intra analyses of both groups revealed that the fatigue domain represented the greatest difference (p<0.05, Table 2). Patients who had a positive energy balance together with adequate protein requirements exhibited a higher HRQoL score (4.9) than those who only ingested the required protein (4.3). No significant differences, between group, were observed (p<0.05). 

**FIGURE 2:**
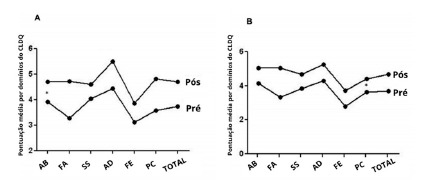
Average CLDQ domain score before and after protein supplementation

WP=whey protein group; C=casein group; Pre=before the supplementation; Post=after supplementation; ABM= abdominal symptoms; FAM=fatigue SYM=systemic symptoms; ACM=domain activity; EMM=emotional function; WOM= worry. * p<0.05

Throughout the study, protein intake among patients in the WP group increased to 1.4±0.6 g/kg, similar to the C group (p>0.05). Although most patients ingested the recommended allowance of protein, the majority still presented a negative energy balance (-424.5±584.9 kcal for the WP, and -350.6±607.3 kcal for the C group, p>0.05). 

There were no changes in the anthropometric measures or in the handgrip strength after the supplementation (p>0.05). The mean 6-minute walking test was similar before and after supplementation (p>0.05).

**TABLE 2:** Results of CLDQ by domains before and after protein supplementation

Group	Domains	Mean	Delta	p
WP	Pre ABM	4.3	1.0	p>0.05
	Post ABM	5.3		
	Pre FAM	3.5	1.3	p< 0.05
	Post FAM	4.8		
	Pre SYM	3.8	1.0	p< 0.05
	Post SYM	4.8		
	Pre ACM	5.0	1.0	p< 0.05
	Post ACM	6.0		
	Pre EMM	3.4	0.6	p< 0.05
	Post EMM	4.0		
	Pre WOM	4.0	1.0	p< 0.05
	Post WOM	5.0		
	Pre TOTAL	4.0	1.0	p< 0.05
	Post TOTAL	5.0		
Group	Domains	Medium		p value
C	Pre ABM	4.0	1.3	p< 0.05
	Post ABM	5.3		
	Pre FAM	3.4	1.6	p< 0.05
	Post FAM	5.0		
	Pre SYM	4.0	0.8	p< 0.05
	Post SYM	4.8		
	Pre ACM	4.1	1.5	p< 0.05
	Post ACM	5.6		
	Pre EMM	3.0	0.9	p< 0.05
	Post EMM	4.0		
	Pre WOM	3.7	1.2	p=0.115
	Post WOM	4.9		
	Pre TOTAL	3.8	1.1	p< 0.05
	Post TOTAL	4.9		

WP=whey protein group; C=casein group; Pre=before the supplementation; Post=after supplementation; ABM= abdominal symptoms; FAM=fatigue SYM=systemic symptoms; ACM=domain activity; EMM=emotional function; WOM= worry

## DISCUSSION

The quality of life of patients with advanced liver disease awaiting transplantation improved after whey protein and casein supplementations. Protein supplementation has been previously associated with an improved HRQoL in malnourished individuals with gastrointestinal diseases ^[^ ^23^ ^]^, chronic heart failure as well as in sarcopenic elderly adults ^[24, 25]^.

To our knowledge, no previous study has evaluated the short-term effects of WP supplementation and the resulting HRQoL in cirrhotic patients waiting for liver transplantation. In this population, studies have been limited to branched-chain amino acid or nutritional counseling alone ^[^ ^26^ ^]^. Kawamura et al., 2004 ^[^ ^27^ ^]^ evaluated the effect this amino acid supplementation on the HRQoL of 25 patients with hepatic cirrhosis during six months. Patients in the amino acid group presented improved HRQoL when compared to those in the control group. According to the authors, this improvement in the HRQoL could be attributed to the decrease of secondary symptoms, such as edema and ascites. Nonetheless, the comparison of our results to these studies is difficult and is further limited by the fact that none used the CLDQ to assess the HRQoL. The advantage of using this questionnaire over other generic questionnaires is its specificity for assessing liver disease. The CLDQ is the only instrument developed for all etiologies and degrees of severity in liver disease, and it was validated for the Brazilian population ^[^ ^28^ ^]^.

Poor HRQoL was associated with a deficient nutritional status, since 64.7% of those with scores <5 were classified as moderately or severely malnourished. Malnutrition in end-stage liver disease patients is multifactorial, a consequence of early satiety due to ascites and portal hypertension, appetite loss and poor nutrient intake (often related to unnecessary salt and protein restriction), as well as the use of various medications ^[^ ^29^ ^]^. Most patients in our study had inadequate energy and protein intake. At study baseline, only 16.0% and 21.4% of the WP patients received adequate energy and protein intake, respectively. We had previously reported in a study conducted with 73 pre-LT patients that more than 80% of our patients had insufficient energy intake ^[^ ^7^ ^]^. In another study conducted by Ney et al., 2015 ^[^ ^30^ ^]^ 76.0% of the 630 pre-LT patients demonstrated a protein intake below 1.2 g/kg/day. 

We hypothesized that WP supplementation, although for a short-time, would contribute to improving these patients’ nutritional and functional status, due to its higher digestibility and the increased number of branched-chain amino acids, especially leucine, thereby improving their quality of life. However, we observed improvement only in the quality of life. These results are partially in accordance with those by Boulhosa et al ^[^ ^31^ ^]^ who reported the effect of 12 weeks of supplementation of two different proteins (casein and soy protein) on the HRQoL of patients with hepatitis C virus. Similar to our study, both groups presented an increased HRQoL score, measured by the Short Form Health Surveillance (SF-36) questionnaire. The authors showed that the improvement in HRQoL was attributed to an increase in lean mass over the 12 weeks period, which we did not observe with our patients.

Certainly, the short time supplementation is the main factor impacting our nutritional and functional results. In most of the studies, the time of amino acid or protein supplementation was greater than 30 days^[24, 27, 32, 33]^. Also, in our study, although most of the patients reached the recommended amount of protein after supplementation, only 25.0% had a positive energy balance at the end. An adequate supply of both energy and protein is essential for protein synthesis^[^ ^14^ ^]^, and nutritional and functional status improvement. Finally, it is also important to acknowledge that the instruments we used to assess the nutritional and functional status maybe incapable of diagnosing minor changes. In this regard, metabolic alterations are the first to be improved, followed by functional capacity markers, and only later anthropometric values ^[17, 34]^. 

The impact of emotional factors on HRQoL was observed in a study conducted by Sharif et al. (2005) ^[^ ^35^ ^]^. They evaluated the HRQoL of 100 patients waiting for LT randomly divided into two groups: control and psychoeducational intervention (information regarding liver disease adaptation to chronic diseases relaxation, exercise, diet and side effects of drugs). The results revealed significant differences in all domains of the CLDQ in the group that received the psychoeducational intervention, while there were no statistically significant differences in the control group^[^ ^35^ ^]^. 

Finally, our study has limitations. The short duration of protein supplementation was determined, after the pilot study was carried out, in which patients’ compliance was low, after the second week. Second, the insensitivity of the tools used to detect changes in nutritional status in short periods is a reality of the current clinical available assessment methods. Furthermore, the use of a 24-hour recall to estimate the daily energy/protein intake may not accurately represent the daily intake throughout the study period, but once again, patients’ adherence to daily registers was low.

## CONCLUSION

Our results indicate that acute supplementation with whey protein or casein similarly improves the HRQoL of patients on the liver transplant waiting list.
